# Functional Role of Histidine in the Conserved His-x-Asp Motif in the Catalytic Core of Protein Kinases

**DOI:** 10.1038/srep10115

**Published:** 2015-05-11

**Authors:** Lun Zhang, Jian-Chuan Wang, Li Hou, Peng-Rong Cao, Li Wu, Qian-Sen Zhang, Huai-Yu Yang, Yi Zang, Jian-Ping Ding, Jia Li

**Affiliations:** 1 National Center for Drug Screening and State Key Laboratory of Drug Research, Shanghai Institute of Materia Medica, Chinese Academy of Sciences, 189 Guo Shoujing Road, Shanghai 201203, China; 2 State Key Laboratory of Molecular Biology, Institute of Biochemistry and Cell Biology, Shanghai Institutes for Biological Sciences, Chinese Academy of Sciences, 320 Yueyang Road, Shanghai 200031, China; 3 School of Life Sciences, East China Normal University, 3663 North Zhongshan Road, Shanghai 200062, China; 4 Drug Discovery and Design Center, State Key Laboratory of Drug Research and Chinese Academy of Sciences Key Laboratory of Receptor Research, Shanghai Institute of Materia Medica, Chinese Academy of Sciences, 555 Zu Chongzhi Road, Shanghai 201203, China

## Abstract

The His-x-Asp (HxD) motif is one of the most conserved structural components of the catalytic core of protein kinases; however, the functional role of the conserved histidine is unclear. Here we report that replacement of the HxD-histidine with Arginine or Phenylalanine in Aurora A abolishes both the catalytic activity and auto-phosphorylation, whereas the Histidine-to-tyrosine impairs the catalytic activity without affecting its auto-phosphorylation. Comparisons of the crystal structures of wild-type (WT) and mutant Aurora A demonstrate that the impairment of the kinase activity is accounted for by *(1)* disruption of the regulatory spine in the His-to-Arg mutant, and *(2)* change in the geometry of backbones of the Asp-Phe-Gly (DFG) motif and the DFG-1 residue in the His-to-Tyr mutant. In addition, bioinformatics analyses show that the HxD-histidine is a mutational hotspot in tumor tissues. Moreover, the H174R mutation of the HxD-histidine, in the tumor suppressor LKB1 abrogates the inhibition of anchorage-independent growth of A549 cells by WT LKB1. Based on these data, we propose that the HxD-histidine is involved in a conserved inflexible organization of the catalytic core that is required for the kinase activity. Mutation of the HxD-histidine may also be involved in the pathogenesis of some diseases including cancer.

Protein kinases are responsible for the regulation of the most prevalent post-translational modification and therefore play vital roles in many essential cellular processes including cell division, cell growth, cell senescence, and cell death. The activities of protein kinases are tightly regulated in eukaryotes and misregulation of kinases is correlated with many diseases^1^,^2^. Since the determination of the first eukaryotic protein kinase (EPK) structure in 1991, numerous kinase structures have revealed the mechanisms underlying the catalysis of protein phosphorylation and the regulation of the kinase activity. In particular, several conserved structure components are recognized to play key roles in the catalysis and regulation of all protein kinases^3^,^4^,^5^,^6^,^7^,^8^,^9^. A typical eukaryotic protein kinase core consists of a smaller N-lobe followed by a larger C-lobe^10^. Generally, ATP and the substrate protein are bound at the substrate pocket located in a cleft between the two lobes. Several conserved components of the N-lobe such as the glycine-rich loop (the G-rich loop) and the Asp-Phe-Gly (DFG) motif are associated with the docking of ATP, while the substrate-binding loop in the C-lobe is responsible for the interaction with the substrate protein. The activation segment located also in the cleft between the two lobes can be auto-phosphorylated or be phosphorylated by upstream kinases. Phosphorylation of the activation segment will trigger the reorientation of the activation segment itself and the catalytic core of kinase. Reorientation of these two elements helps to position ATP and the substrate peptide properly in the catalytic active site, followed by the transfer of the γ-phosphate group of ATP to the substrate and the dissociation of both ADP and the phosphorylated substrate from kinase^3^,^6^. EPKs adopt a closed conformation to phosphorylate the substrates, while they change to an opened conformation when dissociating with the phosphorylated substrates and ADP. The two lobes transform from an opened conformation into a closed conformation to perform a catalytic cycle^9^,^11^,^12^.

Among the elements that are key to sustaining the kinase activity, the HxD triad, which is located in subdomain VI of the kinase catalytic core^13^, is considered to be one of the most highly conserved motifs^5^. The aspartate of this motif is required for the orientation of the hydroxyl group of the substrate peptide at the P-site and the transfer of the phosphoryl group^7^,^14^. Owing to its importance, this aspartate is strictly conserved through all eukaryotic, eukaryotic-like and atypical protein kinases and is one of the most conserved residues in kinases^5^. The second residue in the HxD motif is arginine in most eukaryotic protein kinases; thus, the HxD motif is also called the HRD motif in EPKs, and kinases harboring arginine at this position are defined as RD kinases. In most RD kinases that require phosphorylation of the activation segment, the primary function of the HRD-arginine is to interact with the phosphorylated activation segment, thereby contributing to the correct orientation of the activation segment^7^,^15^. The HxD-histidine is also highly conserved in protein kinases^5^,^13^. In the human kinome, almost all protein kinases harbor histidine at the first position except for a few members of the AGC kinase family in which the HxD-histidine is replaced by tyrosine (Supplementary Fig. S1a-S1b).

In 2005, N. Kannan and A. F. Neuwald suggested that HxD-histidine is a convergence point for catalytic, regulatory and substrate-binding elements because it forms several conserved hydrogen bonds with other key residues in the catalytic core and packs against DFG-phenylalanine^5^. A. P. Kornev *et al.* then demonstrated that the HxD-histidine is part of the regulatory spine (R-spine), a motif comprised of four non-consecutive hydrophobic residues that links the two lobes of kinases^16^,^17^. More recently, K. Oruganty *et al.* found that the backbone of the HxD motif maintains a strain geometry in the active conformation, and the side chain of the HxD-histidine is involved in a critical hydrogen bond network^18^. Although these representative researches have noticed the importance of this highly conserved histidine, its function(s) is still less well understood. Additionally, the questions of whether the HxD-histidine/YxD-tyrosine can be replaced and whether the two types of residues play distinct roles in kinase activity have not yet been addressed.

Here we provide insights into the functions of the HxD-histidine by comparing the activities and crystal structures of wild-type (WT) and HxD-histidine mutant kinases. The function of the HxD-histidine is also partially revealed in our model. We propose that the HxD-histidine is involved in a conserved inflexible organization of the catalytic core which contributes to the accommodation of the active conformation. Mutation in the HxD-histidine, such as the H174R mutation in LKB1 may be involved in the pathogenesis of cancer.

## Results

### The conserved HxD-histidine is irreplaceable for the maintenance of kinase activity

**Figure 1:**
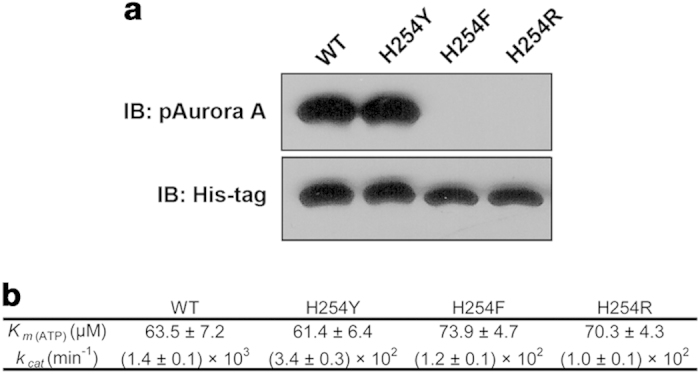
The HxD-histidine is required for the maintenance of the kinase activity of Aurora A. (**a**) Western blot of WT Aurora and the HxD-histidine mutants incubated with ATP. The results are representative of three independent experiments. The catalytic efficiency was also significantly affected by HxD-histidine mutations (**b**) The Michaelis constant *K*_m_ and the maximum rate *V*_max_ was calculated by fitting the Michaelis-Menten model using Prism GraphPad^TM^ software. *k*_cat_ was calculated by fitting *k*_cat_ = *V*_max_ / [*E*]. Where *V*_max_ is the maximum rate, [*E*] is the enzyme concentration. Representative data in three parallel experiments are shown (N = 3).

By comparing the sequences of different members of the human kinome and protein kinase homologs from different organisms, it can be concluded that the HxD-histidine is evolutionarily conserved in all human protein kinases except for a few members of the AGC kinase family (Supplementary Fig. S1a-S1b). Moreover, superposition of the HxD motifs in 44 crystal structures of activated EPKs reveals a conformational conservation in activated protein kinases as well (Supplementary Fig. S1c). The conservation of the HxD-histidine in primary and three-dimensional structures suggests a critical functional role for this residue. We chose Aurora A as a model to examine this hypothesis. In order to investigate which property of the histidine is required for kinase activity, both hydrophobic mutations (H254Y and H254F) and hydrophilic mutation (H254R) were made. Compared with the WT enzyme, the HxD-histidine mutants, i.e. H254Y, H254F and H254R have significantly decreased *k*_*cat*_ with unaffected *K*_*m*_ for ATP (Fig. 1b), indicating that mutation of the HxD-histidine significantly impaired the catalytic activity without affecting the ATP-binding affinity. Interestingly, however, phosphorylation of the activation loop was not affected in the H254Y mutant but was abolished in both the H254F and H254R mutants (Fig. 1a). The *k*_*cat*_ of the Aurora A mutant harboring the Y254RD256 motif was also notably higher than that of other mutants (Fig. 1b). Similar results were also found in several other EPKs (Supplementary Fig. S2). The phenomenon, i.e. different mutations leading to distinguishing extents of activity impairment, suggests that both of the hydrophobicity and hydrophilicity of the side chain of the HxD-histidine are essential to the complete activation of EPKs.

### Mutating the HxD-histidine to either arginine or tyrosine does not affect the overall conformation of Aurora A

**Figure 2:**
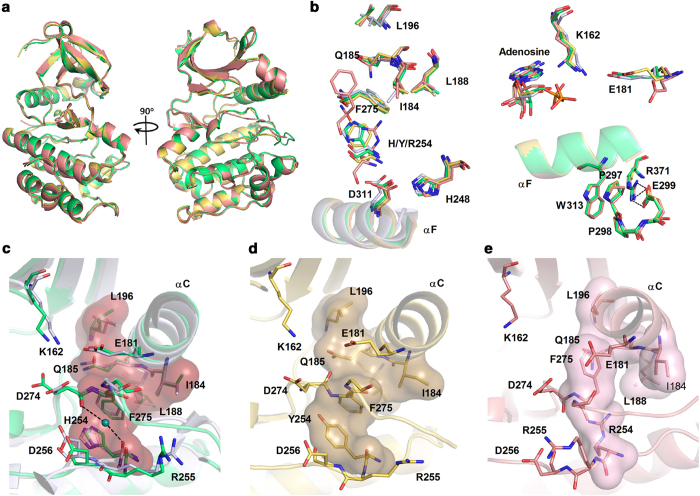
The conserved pattern of the catalytic core and the R-spine are disrupted by the HxD-histidine mutation. (**a**) Overall structure comparison of WT Aurora A and the HxD-histidine mutants. (**b**) Structural alignment of key elements that define the active conformation of EPK. For comparison, fully activated conformation of Aurora A (with phosphorylated activation segment, PDB code: 1MQ4) is used to align with our WT Aurora A and the mutants. Both our WT Aurora A and the H254Y mutant superpose well to the fully activated Aurora A, while the R-spine is disrupted in H254R mutant. (**c**-**e**) Detail views of the critical residues in the catalytic cores of WT Aurora A, the H254R and the H254Y mutants. The carbons of the WT, H254R and H254Y Aurora A proteins are colored green, pink and yellow, respectively. The fully activated conformation of Aurora A is colored gray. R-spine and the hydrophobic hook are shown as surface model. The conserved water molecule in the catalytic core of the activated Aurora A is shown as blue sphere. Polar interactions are shown as black dash lines.

To investigate the mechanisms by which HxD-histidine mutations interfere with the kinase activity of Aurora A, we determined the crystal structures of WT Aurora A and the H254R and H254Y mutants (Glu122-Ser403). The summary of data collections are listed in Table 1. Given that the HxD-histidine mutants except H254Y cannot be phosphorylated and phosphorylation on the activation-segment plays critical roles in arrangement of the catalytic cores of protein kinases, adenosine was used to co-crystalize with the proteins to obtain a more appropriate un-phosphorylated control to analyze the effects of the HxD-histidine mutations. Since many studies have reported at length about the bilobal structure of EPKs, here we will only discuss new insights derived from our structural data. Unlike the previously solved inactive structure of the same complex^19^ (PDB code: 1MUO), identification of an assembled R-spine fixed on helix-F indicated that we obtained crystal of WT Aurora A–adenosine complex with an active conformation (Fig. 2b,c). Meanwhile, the packaged W313-P297-P298 and the salt-bridge between E299 and R371 in our WT structure also serve as hallmarks of active conformation of EPKs^20^ (Fig. 2b). Structural alignment of the two mutants to the WT enzyme shows that mutation of the HxD-histidine to either tyrosine or arginine does not affect the overall conformation of Aurora A (Fig. 2a). Major impact of the HxD-histidine mutation is on the configuration of several residues around the catalytic core.

### Hydrophilic mutation of the HxD-histidine severely disrupted the conserved pattern of the catalytic core

**Figure 3:**
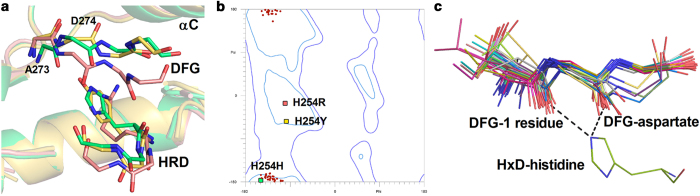
The HxD-histidine is involved in the preservation of the active conformation of xDFG motif. (**a**) Conformational changes of the HxD- and xDFG-backbones in the H254R and H254Y mutants. Only the side chains of H/R/Y254 are shown as stick models for clarity. The carbons of the WT, H254R and H254Y Aurora A proteins are colored green, pink and yellow, respectively. (**b**) Ramachandran plot shows the conserved conformation of the xDFG-backbone in active EPKs. Dihedral angles of the DFG-1 residues in the active conformations listed in Table S1 are indicated as red dots. Dihedral angles of A273 in WT as well as the H254R and H254Y mutants are indicated as green, pink and yellow squares, respectively. (**c**) Superposition of the xDFG-backbones of the active structures listed in Table S1. For clarity, only the HxD-histidine of Aurora A is shown in line model. Hydrogen bonds between the HxD-histidine and xDFG-backbone are shown as black dash lines.

In WT Aurora A, the HxD-histidine is positioned in the center of the catalytic core, connecting and bracing the key residues of the catalytic core (Fig. 2c). Although the complex of WT enzyme and adenosine cannot be auto-phosphorylated, conformation of the catalytic core in our WT structure is closely similar with that in the completely activated structure of WT Aurora A (PDB code: 1MQ4, Fig. 2b,c, RMSD = 0.257). This conserved conformation can be defined as “the conserved pattern of the catalytic core”. Besides, the π-π conjugation between the side chains of H254 and F275, as well as the interactions among the F275, Q185 and L196 compose an intact R-spine of Aurora A. Hydrophobic interactions among F275, I184 and L188 also contribute to anchor the N-terminus of the activation segment^6^. In the H254R mutant, however, these conserved activated conformations are severely disrupted (Fig. 2d). Aromatic ring of F275 is flipped into a “DFG-Phe-out” conformation, which not only disorders the backbone conformations of the HxD and DFG motifs (Fig. 3a), but also cripples the integrity of the R-spine (Fig. 2b). The hydrophobic anchor at the N-terminus of the activation segment is also abolished by the shift of the DFG-phenylalanine. Moreover, the out-flipped phenyl ring of F275 is inserted into the space between K162 and E181 (Fig. 2d). The active conformation of EPKs is characterized by a conserved salt-bridge between a lysine (K162 in Aurora A) on strand β3 and a glutamate (E181 in Aurora A) on helix αC, through which helix αC is tightly connected to the rigid main body of the N-lobe^21^. Therefore, the disruption of the Lys-Glu contact, the dissociation of the R-spine, and the hydrophobic anchor of the activation segment N-terminus conspire to inactivate the H254R mutant.

### The HxD-histidine is critical to preserve both the active conformations of the DFG-1 residue and the DFG motif

Distinct from the H254R mutant, the H254Y mutant-adenosine complex exhibits an active configuration similar with the WT structure (Fig. 2b,e). In the H254Y mutant, the conformations of K162 and E181 as well as the integrity of the R-spine are all unaffected. Replacement of histidine by tyrosine in the HxD motif retained the π-π interaction with the DFG-phenylalanine due to the similarity between the histidine and tyrosine side chains. The orientation of the side chain of Y254 in the H254Y mutant is also similar to that of H254 in the WT (Fig. 2b and 3a). However, our structural models still identify some significant conformational changes in the catalytic core of the H254Y mutant. Although the electrostatic properties of the side chains of His and Tyr are similar to a certain extent, conformation of the main chain of the DFG motif in the H254Y mutant is different from that of the WT enzyme (Fig. 3a). A flip-over conformation of the A273 backbones is clearly defined by the electron density of the two mutants (Fig. 3a,b, Supplementary Fig. S3). Orientation of the main-chain carbonyl of D274 in the H254Y mutant also has to be replaced overturned in refinement procedures, on account of the larger steric hindrance caused by the side chain of tyrosine than that caused by histidine, although electron density maps cannot define the orientation clearly due to the moderate resolution. The conserved unfavorable conformation of the backbone of the DFG motif is considered to be correlated with EPK activity^18^,^21^. Moreover, as shown in Fig. 3b, comparison of 44 high-resolution structures of EPKs indicates that the torsion angles of the residues preceded the DFG motifs are highly similar in active conformations of EPKs. Hydrogen bonds between the HxD-histidine side chains as well as the main-chain carbonyls of the DFG-1 residues and the DFG-aspartates are also highly conserved in these structures (Fig. 3c). Thus we prefer to integrally name the DFG-1 residue and the DFG triad as xDFG motif. Flipping of the peptide plane between A273 and D274, as well as the extremely possible turnover of the main-chain carbonyl of D274, not only loosen the unfavorable conformation of the xDFG-backbone, but also abolish the conserved hydrogen bonds between the side chain of the HxD-histidine and the main-chain carbonyls of the xDFG motifs. Therefore, these inactive conformational features interpret the H254Y mutant as partial inactivation, which is consistent with our activity assay (Fig. 1b).

**Figure 4:**
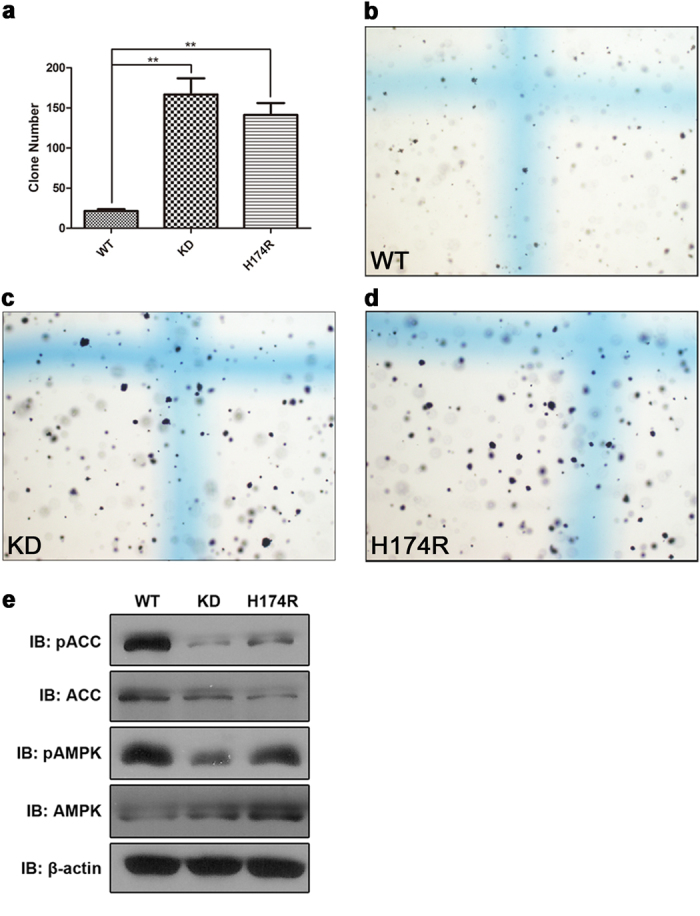
Mutation of the HxD-histidine in the tumor suppressor LKB1 kinase impaired its activity and the ability to suppress anchorage-independent growth. A549 cell lines over-expressing WT, K87I (KD mutant), or H174R LKB1 were analyzed for their ability to form colonies in agarose (**a**-**d**). The average numbers of colonies per six-well plate (N = 3) were counted after 14 days. The error bars represent the S.E.M. Significance was analyzed using a two-tailed unpaired Student t test. A value of *p* < 0.01 (^**^) was considered highly statistically significant. (**e**) Immunoblotting analysis of the active LKB1-AMPK pathway by detection of phospho-AMPK and phospho-ACC in A549 cells over-expressing WT LKB1, H174R, or K87I mutants. Representative results of three independent experiments are shown.

Somatic mutation of the HxD-histidine in tumor suppressor LKB1 kinase impairs its activity and ability to suppress anchorage-independent growth of A549 cells. Both loss-of-function and gain-of-function mutations in kinases have been demonstrated to play critical roles in tumorigenesis. To investigate whether HxD-histidine mutations exist in human cancers, we searched COSMIC and dbSNP for Single Nucleotide Polymorphisms (SNPs) associated with residues around the catalytic loop of kinases serving as tumor suppressors. The databases showed that HxD-histidine mutants of two tumor suppressors, LKB1 (H174R, COSM mutation ID: COSM27283) and tyrosine kinase 2 (TYK2, H1021Y, COSM mutation ID: COSM20405) have been found in the tumor tissues of lung cancer^22^ and large intestine cancer patients, respectively. Besides, the LKB1 harboring H174R mutation was also identified in colon polyps of a male patient with colon carcinoma^23^. The loss-of-function of LKB1 has been particularly well studied in lung cancer^24^,^25^,^26^,^27^,^28^. To further investigate the biological functions of the HxD-histidine and its relationship with carcinogenesis, the H174R mutant of human LKB1 and its kinase-dead mutant (K78I) were obtained and transfected into the LKB1-deficient A549 cell line to construct stable cell lines. These cell lines were evaluated with colony formation assays. Cells overexpressing either of the mutants had stronger colony-forming abilities than cells overexpressing WT LKB1, suggesting that similar to the kinase-dead mutation, the H174R mutation of LKB1 abolishes the tumor-suppressing ability of LKB1 (Fig. 4a-d). Because phosphorylation of AMPK is an event occurs downstream of LKB1 activation, the phosphorylation levels of AMPK αT172 and its well-known downstream target ACC were also examined to evaluate the activity of LKB1. As shown in Fig. 4e, the phosphorylation levels of both AMPK and ACC were significantly reduced in A549 cells overexpressing the H174R or K78I mutant, which is in good agreement with the results of the colony formation assays.

## Discussion

All kinases have similar tertiary structures composed of several highly conserved core components, and their catalytic mechanisms evolved through elaboration of these conserved components. Optimal catalysis requires the proper assembly of two lobes. In the optimal orientation of the N-lobe, it is particularly important that the G-rich loop and helix αC are correctly positioned. The G-rich loop must be optimally positioned to allow the proper binding of ATP and the transfer of the γ-phosphate^17^, whereas helix αC should be properly oriented to interact with the activation segment. The HxD-histidine, which is replaced by tyrosine in only a few kinases of the AGC kinase family, is one of the most conserved critical residues shared by almost all members of EPKs and eukaryotic like kinases (ELKs) and has been suggested to play a role in accommodation of the two lobes of kinases 5,18. In the active conformations of kinases, the HxD-histidine and the YxD-tyrosine adopt similar orientations (Supplementary Fig. S1c). It is convinced previously that the importance of the HxD-histidine is due to its involvement in the assembly of the R-spine^3^,^4^,^5^,^16^,^17^,^18^. Here, the comparison between the structures of WT Aurora A and its HxD-histidine mutants reveals that the HxD-histidine may be involved in a more complicated interaction cascade than that which was noted previously.

The inactive structure of the H254R mutant well demonstrate the importance of the hydrophobic interact between the HxD and xDFG motif. In the H254Y mutant of Aurora A, however, the π-π interaction between the mutated HxD motif and the DFG-phenylalanine is retained, preserving the integrity of the R-spine and the hydrophobic anchor at the N-terminus of the activation segment. However, intriguingly, the kinase activity of Aurora A was significantly abrogated by the H254Y mutation, although its autophosphorylation was not affected. A recently published research also reported that the H254Y mutation do not affect the autophosphorylation of Aurora A^18^. Although this study shows that, similar to the WT kinase, the H254Y mutant can also phosphorylate histone H3, the authors did not evaluate the catalytic velocity of these two enzymes. In our assay, however, the H254Y mutant showed significantly impaired catalytic efficiency compared with the WT enzyme. Although the N-terminal 121 residues were deleted in both the WT and the mutated Aurora A for better protein expression, the kinase activity of the WT Aurora A was not affected by the truncation (Supplementary Fig. S4). Furthermore, the loss of kinase activity due to the H254Y mutation is in accordance with the conformational change observed in the crystal structure of the H254Y mutant, i.e. the loss of the strained conformation of the xDFG-backbone as well as the disruption of the hydrogen bond network connecting the activation segment and the xDFG motif. The mechanism of autophosphorylation is still disputed nowadays. Dodson *et al.* interpreted the autophosphorylation mechanism of Aurora A as an intramolecular way^29^, whereas Zorba *et al.* reported an intermolecular autoactive manner through an asymmetric domain-swapped dimmer^20^. Electrostatics analyses reveal that the high electric potential energy around the RD pocket is abolished in the H254R mutant, but it is not affected in the H254Y mutant (Supplementary Fig. S5). This result may explain why autophosphorylation of the H254Y mutant was unaffected when the autophosphorylation mechanism of Aurora A is intramolecular since an alkalic RD pocket is required for this mechanism, while the intermolecular autoacitvation is also rational because of the moderate activity of the H254Y mutant. Rather, the H254R mutant cannot autophosphorylate itself through any of these mechanisms.

**Figure 5:**
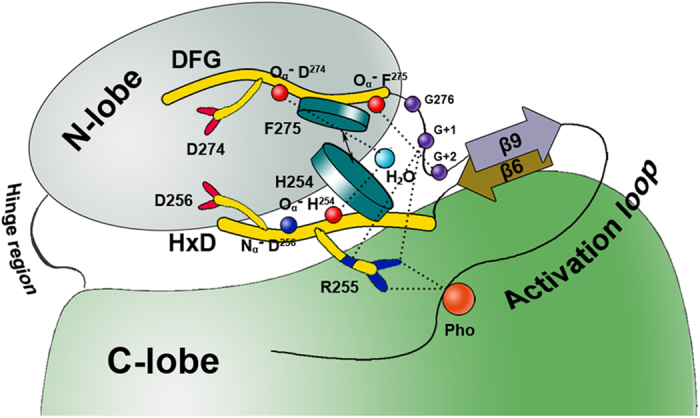
A schematic diagram showing the conserved inflexible organization of the catalytic core (Aurora A is used as the example). This pattern is conserved through the active structure listed in Table S1. The hydrophobic interaction between the HxD-histidine and the xDFG-phenylalanine packages up the HxD and the xDFG motifs. The hydrogen bonds among the HxD-histidine, the xDFG-backbone, and the conserved water molecule in the catalytic core are involved in the conformational maintenance of the catalytic core. The hydrogen bonds among the HxD-arginine, the xDFG-aspartate, G + 1 and G + 2 residues have been considered to be required for the catalytic activity. Carbons in the HxD and the xDFG motifs are colored yellow. The key atoms in the HxD and xDFG motifs are shown as sphere models. The G276, W277 and S278 residues are shown as purple spheres. The conserved water molecule and the phospho-site are shown as blue and orange spheres, respectively.

The severe disruption of the active conformation caused by the hydrophilic mutation of the HxD-histidine demonstrates that the hydrophobicity of this conserved residue is essential to the catalytic activity of EPKs. On the other hand, although a recent research using PKA as model had shown that hydrophobic mutations of YxD-tyrosine, including Tyr-to-His mutation, had relatively slight impacts on the phosphorylation of the activation segment^30^, the significant decrease of the catalytic velocity of the H254F and H254Y mutants also indicate other irreplaceable roles of the HxD-histidine except participating in the formation of the R-spine. Substitution of histidine by tyrosine in the HxD motif leads to the shift of the xDFG-backbone conformation because of the steric hindrance. For the reason that the affinities of the kinases for ATP are all affected by magnesium^31^, and the *K*_*m*_ values of the WT Aurora A and those of the mutants have no significant difference, the conformational change of the xDFG-backbone should have no impact on the magnesium binding of the mutant. Thus, the activity loss of the H254Y mutant should be attributed to the interruption of the hydrogen bond network around the catalytic core (Fig. 5). In this network, both of the hydrogen bonds between the side chain of the HxD-histidine and the main chain carbonyls of the DFG-1 residue and the xDFG-aspartate are likely involved in the preservation of the backbone conformations of the xDFG motif. The fact that this network can be disrupted by a single point mutation of the HxD-histidine indicates that the organization of the residues in the catalytic core of EPKs is relatively compact and inflexible. Hydrophobic interaction between the HxD-histidine and the xDFG-phenylalanine packages the catalytic loop and the magnesium-binding loop together, while hydrophilicity of the side chain of the HxD-histidine participates and stabilizes the hydrogen bond network around the catalytic core. Stabilization of this network facilitates the formation of the conserved configuration of the catalytic core and vice versa. In the H254Y mutant, the steric hindrance caused by the mutated tyrosine side chain impedes the formation of the inflexible organization of the catalytic core. Therefore, it is the amphiphilicity and appropriate size of imidazole ring that render the HxD-histidine the ability to unite the residues around the catalytic core together.

Since the coordination of ATP and Mg^2+^, in which the xDFG motif is involved, may subtly change the conformation of the catalytic core, to obtain crystal structures of the mutants with authentic ATP substrate bound will be very helpful to elucidate the function of the HxD-histidine more convincingly. Nonetheless, both the kinetic and structural data demonstrate that mutation of the HxD-histidine causes kinase inactivation. The influences of this mutation on cellular function were also observed in our study. Similar to other somatic mutations of key residues^32^,^33^,^34^,^35^, SNPs of the HxD-histidine could also significantly affect the biological functions of kinases. Our data suggest for the first time that the H174R mutant of LKB1 has an important role in tumorigenesis. It has been well documented that as a tumor suppressor, LKB1 is a critical inhibitor of pulmonary tumorigenesis^24^,^26^,^27^,^36^, and loss-of-function somatic mutations in LKB1 are frequently detected in human lung adenocarcinomas^25^,^28^,^35^. Among these mutations, Y49D and G135R have been shown to be cancer driver mutations^37^. Here, we suggest that the H174R LKB1 mutant also has decreased kinase activity and tumor suppressing capability in A549 cells, compared with WT. It has been reported that LKB1 inhibits lung cancer progression through lysyl oxidase and extracellular matrix remodeling^26^ and down-regulation of the PI3K/PTEN pathway^36^. Catalytically deficient LKB1 mutants can also enhance the expression of the oncogene cyclin D1 through recruitment to the cyclin D1 promoter^38^. The H174R LKB1 mutant may also play a role in pulmonary tumorigenesis via these mechanisms. Although the crystal structure of the heterotrimer of LKB1 holoenzyme reveals a unique mechanism involved in the activity regulation process of the complex^39^, the catalytic domain of active LKB1 still adopts a conformation as canonical as Aurora A. The conserved pattern of the catalytic core and the intact regulatory spine are both presented in the configuration of LKB1 (Supplementary Fig. S6), despite the conserved phenylalanine of the DFG-motif is replaced by a leucine. The similar organizations of the catalytic domain between the Aurora A and LKB1 suggested that the similar inactive mechanisms are probably involved in the H254R mutant of Aurora A as well as the H174R of LKB1.

Together, our data reveal the mechanisms by which the HxD-histidine is involved in the maintenance of the conserved inflexible organization of the catalytic core. These mechanisms and the involvement of the HxD-histidine mutant in tumorigenesis may aid the design of kinase inhibitors and drug-discovery efforts for cancer treatment.

## Experimental Procedures

### Expression and Purification of WT and Mutant Aurora A Proteins

The gene encoding WT human Aurora A was obtained from Addgene (plasmid NM_003600). The gene fragment encoding the truncation of Aurora A encompassing residues 122-403 was cloned into the NcoI/Xhol sites of a pET-28b vector which adds an N-terminal 6×His-tag to the protein. The gene encoding full length WT human ERK1 and WT human GSK3β were cloned into pGEX-KG vectors. The gene fragment encoding the kinase domain of WT human AMPKα2 subunit (1-285) was cloned into a pET-28a vector. Mutations were introduced using the Stratagene QuikChange II kit. *Escherichia coli* BL21 (DE3) pLysS cells (obtained from Shanghai Institute of Biochemistry and Cell Biology) transformed with the plasmid were grown at 37 °C until OD reached 0.4 ~ 0.6, and induced with isopropyl-β-D-thiogalactopyranoside at a final concentration of 0.2 mM at 22 °C (for Aurora A), 20 °C (for GSK3β), or 18 °C (for AMPK and ERK1) for further 10 hr. Cells were harvested by centrifugation. Cells expressing 6×His-tagged proteins and GST-tagged proteins were resuspended in buffer A (20 mM Tris-HCl, pH 8.0) and PBS, respectively. The suspensions were then lysed by sonication on ice. After centrifugation, the resultant supernatants containing 6×His-tagged proteins were loaded onto the Ni-NTA agarose (Qiagen, Hilden, Germany), which was pre-equilibrated with buffer A. Then the 6×His-tagged proteins were eluted with an elution buffer (buffer A supplemented with 250 mM imidazole). The supernatants containing GST-tagged proteins were loaded onto the GST resin pre-equilibrated with PBS, and the proteins were eluted by PBS containing 10 mM GSH. Purified proteins were desalted by dialyzing in buffer B (20 mM Tris-HCl, pH 7.5, 150 mM NaCl, 5% glycerol and 1 mM DTT).

### Crystallization and diffraction data collection

Co-crystallization of the proteins with adenosine was performed at 4 °C using the hanging drop vapor diffusion method. Bean-shaped crystals were grown in drops containing equal volumes (2 μL) of the protein mixture solution and the reservoir solution (0.1 M Bis-Tris, pH = 5.5, 0.2 M Ammonium sulfate, 25% w/v PEG 3350) to the maximum size in 1–2 days. Diffraction data were collected from flash-cooled crystals at −176 °C at beamline BL-17U in the Shanghai Synchrotron Radiation Facility (Shanghai, China) and processed with HKL2000^40^ subsequently. The structure was solved with the molecular replacement method implemented in the program suite CCP4 by using the structure of the human Aurora A kinase (PDB code 1MQ4) as search model. The initial structure refinement was carried out with program Phenix^41^ and REFMAC5^42^. Model building was performed manually with the program Coot^43^. Throughout the refinement, 5% of randomly chosen reflections were set aside for calculating free *R* value. The final stereochemical quality of structural models was checked with MolProbity^44^. A summary of structure refinement is listed in Table 1.

### Western blots and kinase activity assay

Various amounts of WT or mutant Aurora A proteins were added into the kinase buffer (20 mM Tris-HCl pH 8.0, 5 mM MgCl_2_, and 1 mM DTT) at a final concentration of 200 nM and then incubated with 200 μM ATP at 30 °C for 1 hr. The samples were then loaded on 12% (wt/vol) SDS-PAGE and immunoblotted using an anti-phospho-Aurora (T288) antibody (Cell Signaling) and an anti-His-tag antibody (Cell Signaling). The bands were visualized using HRP-conjugated secondary antibody and ECL substrate (Thermo).

The kinase dynamic assays of Aurora A were performed using the ADP Hunter^TM^ Assay Kit (Discoverx). 20 μL of the reaction mixture, which contained either 6 μM recombinant histone H3, 200 nM WT Aurora A or the H254Y mutant, or 6 μM recombinant histone H3, 10 μM H254R mutant or the H254F mutant, 10 μL Reagent A and 20 μL Reagent B were added into 384-well plate. Fluorescence were read in real-time using EnVision^TM^ (PerkinElmer). The Michaelis constant *K*_m_ and the maximum rate *V*_max_ was calculated by fitting the Michaelis-Menten model using Prism GraphPad^TM^ software. *k*_cat_ was calculated by fitting *k*_cat_ = *V*_max_ / [*E*]. Where *V*_max_ is the maximum rate, [*E*] is the enzyme concentration.

The activities assays of ERK1 and GSK3β were performed with Z-lyte^TM^ Assay Kit (Invitrogen). 5 μL reaction mixtures contained 1 μM substrate peptide (SP) and 20 μM ATP, as well as variant concentrations of the mixtures of ERK1 and MAK (at molar ratio of 1:5), or 5 μL mixtures contained variant concentrations of GSK3β, 1 μM substrate peptide (SP) and 30 μM ATP, were added into 384-well plate and were incubated for 1 hour at 30 °C. Fluorescence were read in EnVision^TM^ after 2.5 μL developmental buffer and 2.5 μL stopping buffer were added into each well. The activity assays of AMPKα2 were performed by the htrf^TM^ Assay Kit (cis-bio). 200 nM AMPKα2 and its mutants was pre-phosphorylated in reaction buffer containing 80 nM LKB1, 20 mM Tris-HCl pH = 8.0, 5 mM MgCl_2_, 1 mM DTT and 200 μM ATP for 2 hours at 30 °C. 6 μL reaction mixtures containing variant concentrations of pre-phosphorylated AMPK, 200 μM ATP and substrate peptide, were added into 384-well plate followed by incubation at 30 °C for 15 min. Fluorescence were read in EnVision^TM^ after 5 μL detection buffer was added into each well.

### Cell culture and colony formation assays

A549 cells were obtained from ATCC. For soft agar colony formation assays, A549 stable cell lines expressing WT LKB1, LKB1-KD (K78I), or H174R mutants were suspended in F12 culture medium containing 10% FBS and 0.3% agarose. Cells were seeded at 2000 per well onto a layer of F12 culture medium containing 10% FBS and 0.8% agarose in 12-well plates, allowed for proliferation for 14 days, and stained with 0.5 mg/mL nitrotetrazolium blue chloride (Sigma-Aldrich) for 24 hr. Colonies were visualized by light microscopy (4×) and counted. Results were expressed as mean values from triplicate wells.

## Additional Information

**How to cite this article**: Zhang, L. *et al*. Functional Role of Histidine in the Conserved His-x-Asp Motif in the Catalytic Core of Protein Kinases. *Sci. Rep.* **5**, 10115; doi: 10.1038/srep10115 (2015).

## Supplementary Material

Supporting Information

## Figures and Tables

**Table 1:** Summary of diffraction data and structure refinement statistics.

	**Aurora A**	**Aurora A**	**Aurora A**
	**WT**	**H254R**	**H254Y**
**Diffraction data**			
Wavelength (Å)	0.9791	0.9793	0.9791
Space group	*P*6_1_22	*P*6_1_22	*P*6_1_22
Cell parameters			
*a* (Å)	82.2	84.4	82.7
*b* (Å)	82.2	84.4	82.7
*c* (Å)	172.6	175.4	172.6
Resolution (Å)	40.0-2.50 (2.59-2.50)^a^	50.0-2.60 (2.69-2.60)	40.0-2.60 (2.69-2.60)
Observed reflections	129,470	224,040	123,484
Unique reflections (I/σ(I) > 0)	12,580	12,050	10,926
Average redundancy	10.3 (10.8)	18.6 (13.4)	11.1 (7.2)
Average I/σ(I)	34.4 (3.3)	37.8 (3.8)	36.8 (3.6)
Completeness (%)	99.4 (99.6)	99.7 (98.8)	96.2 (83.3)
*R*_merge_ (%)^b^	6.6 (67.0)	7.6 (54.6)	6.4 (52.3)

**Refinement and structure model**			
Reflections (*Fo≥0*σ*(Fo*))			
Working set	11880	11432	10333
Test set	650	572	576
*R*_work_/*R*_Free_ (%)^c^	23.7 / 25.0	23.4 / 27.3	24.4 / 27.6
Average B factor (Å^2^)			
All atoms	73.1	70.7	74.2
Protein	73.0	70.8	74.3
Adenosine	105.5	90.5	83.5
Water	59.0	59.1	65.5
RMS deviations			
Bond lengths (Å)	0.006	0.006	0.005
Bond angles (°)	1.02	1.09	1.05
Ramachandran plot (%)			
Favoured	97.7	97.6	97.6
Outlier	0	0	0
PDB Code	4O0S	4O0U	4O0W

^a^Numbers in parentheses represent the highest resolution shell.

^b^Rmerge = ∑_hkl_∑_i_|I_i_(hkl)− < I(hkl) > |/∑_hkl_∑_i_I_i_(hkl).

^c^R = ∑_hkl_||F_o_|−|F_c_||/∑_hkl_|F_o_|.
