# Supplementary material for: Functional Role of Histidine in the Conserved His-x-Asp Motif in the Catalytic Core of Protein Kinases
Source: Sci Rep. 2015 May 11;5:10115. doi: 10.1038/srep10115 (PMC4650784; doi:10.1038/srep10115)
Supplement: Supporting Information [file srep10115-s1.pdf]

Supplementary Information:

Functional Role of histidine in the conserved His-x-Asp motif in the Catalytic Core of Protein Kinases

Lun Zhang[1], Jian-Chuan Wang[2], Li Hou[1], Peng-Rong Cao[1], Li Wu[3], Qian-Sen Zhang[4], Huai-Yu Yang[4], Yi Zang[1,*], Jian-Ping Ding[2,*] and Jia Li[1,3,*]

[1]National Center for Drug Screening and State Key Laboratory of Drug Research, Shanghai Institute of *Materia Medica,* Chinese Academy of Sciences, 189 Guo Shoujing Road, Shanghai 201203, China
[2]State Key Laboratory of Molecular Biology, Institute of Biochemistry and Cell Biology, Shanghai Institutes for Biological Sciences, Chinese Academy of Sciences, 320 Yueyang Road, Shanghai 200031, China
[3]School of Life Sciences, East China Normal University, 3663 North Zhongshan Road, Shanghai 200062, China
[4]Drug Discovery and Design Center, State Key Laboratory of Drug Research and Chinese Academy of Sciences Key Laboratory of Receptor Research, Shanghai Institute of Materia Medica, Chinese Academy of Sciences, 555 Zu Chongzhi Road, Shanghai 201203, China

*To whom correspondence should be addressed: Jia Li, 189 Guo Shoujing Road, Shanghai 201203, China, Tel: 86-21-50801552, E-mail: jli@simm.ac.cn; or Jianping Ding, 320 Yueyang Road, Shanghai 200031, China, Tel: 86-21-54921619, E-mail: jpding@sibcb.ac.cn; or Yi Zang, 189 Guo Shoujing Road, Shanghai 201203, China, Tel: 86-21-50801552, E-mail: yzang@simm.ac.cn;

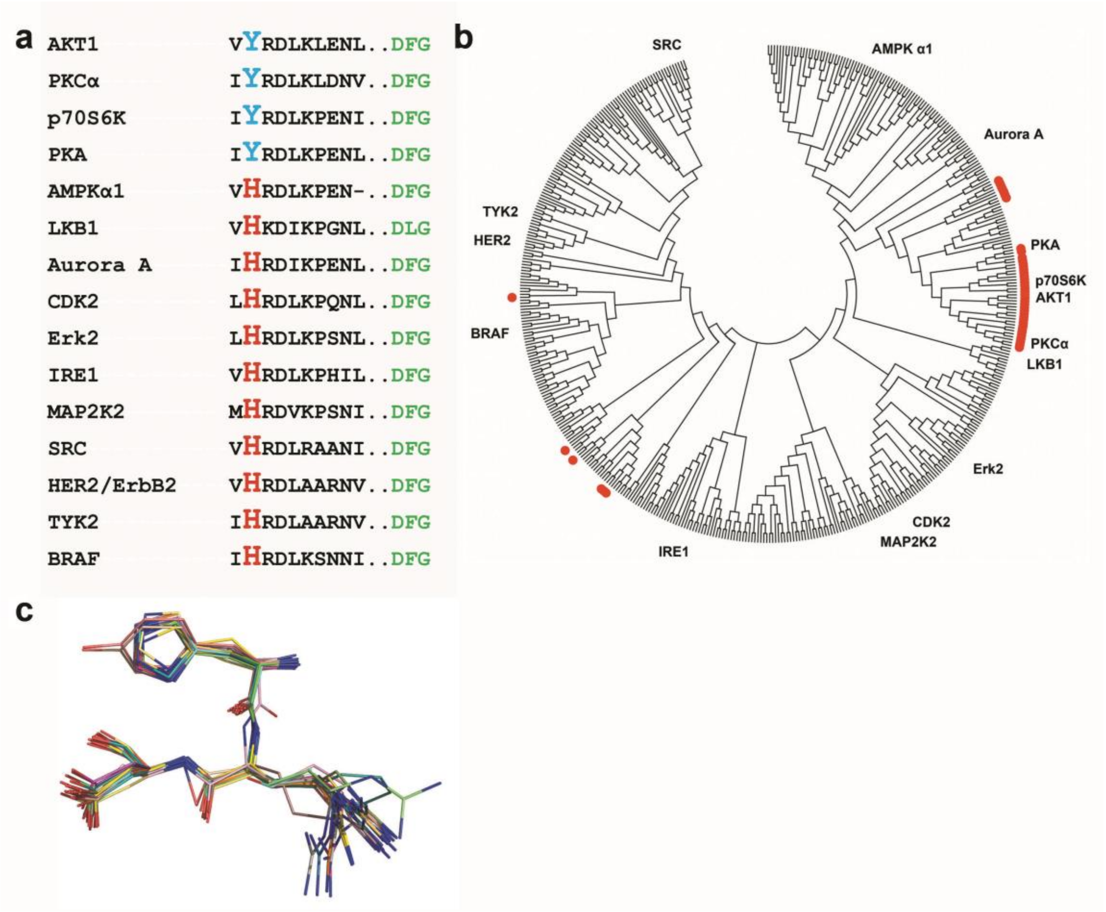

Fig. S1 HxD-histidine is highly conserved in protein kinases. HxD-histidine is conserved through different members of human kinome *(a-b)*. HxD histidine is replaced by tyrosine mainly in partial members of AGC kinase family *(b)*. In spite of the differences in sequences, the conformation of HxD/YxD motif is conserved in activated kinases conformation *(c)*. Kinases aligned in *(a)* are all labeled in *(b)*. 45 YxD-kinases are labeled by red dots in *(b).* Conformations aligned in *(c)* have been listed in Table S1.

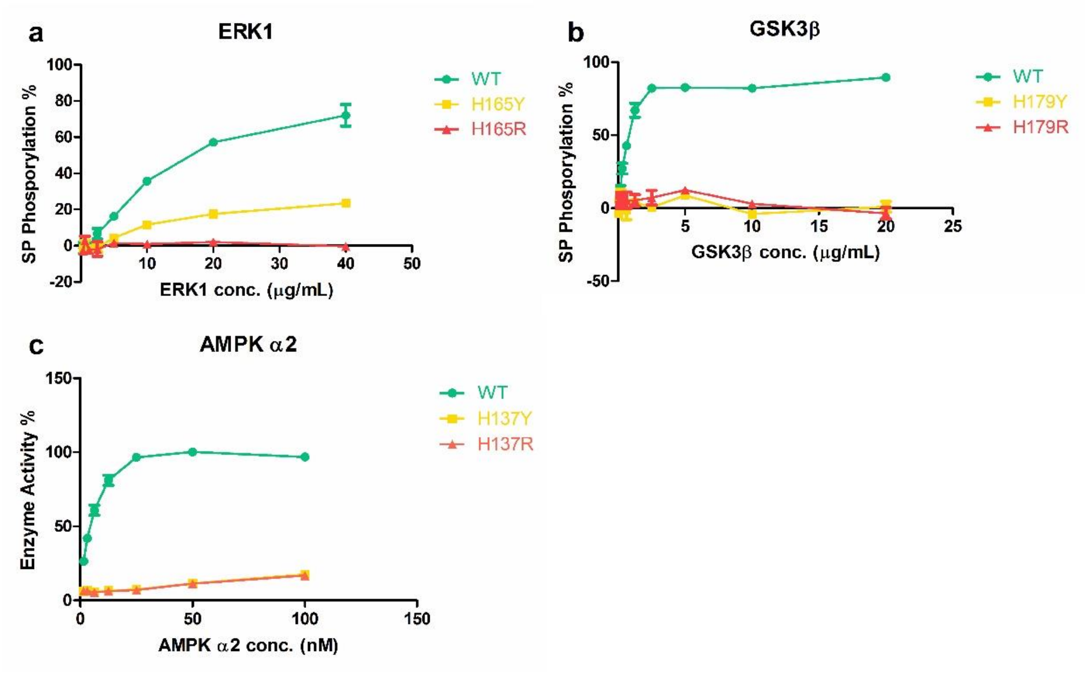

Fig. S2 Kinase activities of EPKs are impaired by the HxD-histidine mutations. *(a-b)* Assays were carried out using Z-lyte™ Assay Kit from Invitrogen. Fluorescence of the reaction mixtures containing 1μM substrate peptide (SP) and 20μM ATP as well as variant concentrations of the mixtures of ERK1 and MAK (at molar ratio of 1:5), or the reactions containing variant concentrations of GSK3β as well as 1μM substrate peptide (SP) and 30μM ATP, were read after the mixtures were incubated for 1 hour at 30˚C. *(c)* Assays were carried out with htrf™ Assay Kit from cis-bio. 200 nM AMPKα2 subunit (1-285) or its mutant were pre-phosphorylated in reaction buffer containing 80nM LKB1, 20 mM Tris-HCl pH=8.0, 5 mM $MgCl_2$, 1 mM DTT and 200 μM ATP for 2 hours at 30˚C. Fluorescence of the reaction mixtures containing variant concentrations of pre-phosphorylated enzymes as well as 200μM ATP and substrate peptide were read after the mixtures were incubated at 30˚C for 15min.

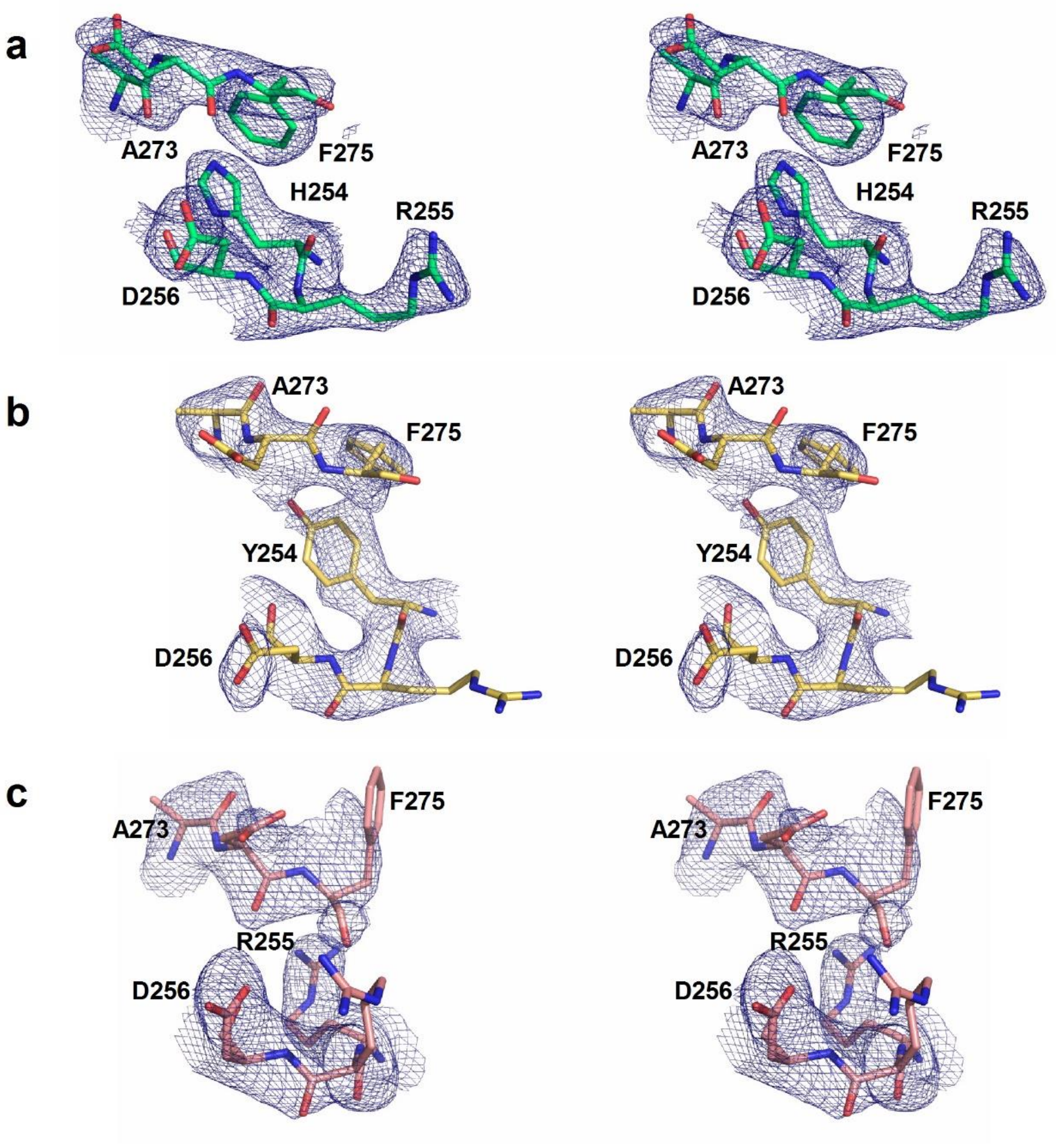

Fig. S3 2mFo-Fc electron density maps of the key residues of the WT *(a),* H254Y *(b)*, and H254R *(c)* Aurora A proteins. Contour level of the 2Fo-Fc maps is 1.0. The carbons of the WT, H254Y and H254R Aurora A proteins are colored green, yellow and pink, respectively.

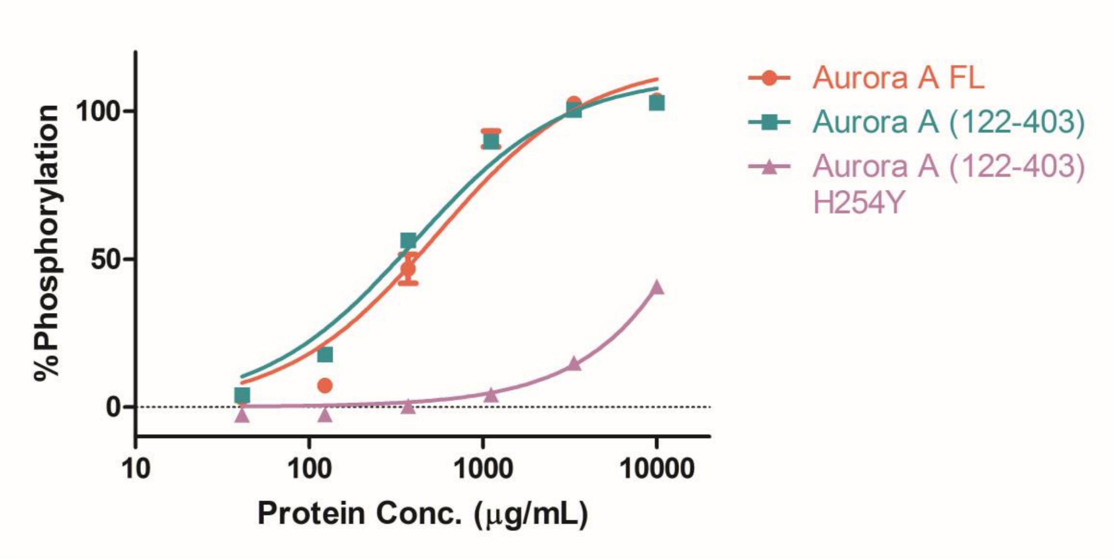

Fig. S4 Kinase activity of Aurora A is not affected by the truncation. Assay was carried out using Z-lyte™ Assay Kit from Invitrogen. 200 nM enzymes were separately incubated in reaction buffer containing 20 mM Tris-HCl pH=8.0, 5 mM $MgCl_2$, 1 mM DTT and 200 μM ATP for 1 hour at 30˚C. Variant concentrations of enzymes were mixed with substrate peptide and incubated for 1 hour at 30˚C.

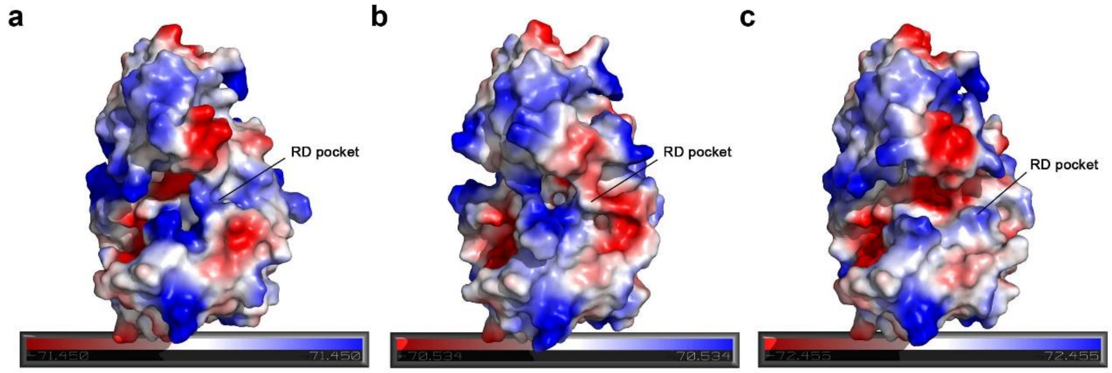

Fig. S5 HxD-histidine is related to the electric potential energy of the RD pocket. Vacuum electrostatics analyses of WT Aurora A's and its HxD histidine mutants' surface charge show that WT Aurora A possessed high electric potential energy around its RD pocket *(a)*. The potential energy is decreased in H254R mutant *(b)*, while H254Y mutant do not change the surface charge of the RD pocket *(c)*.

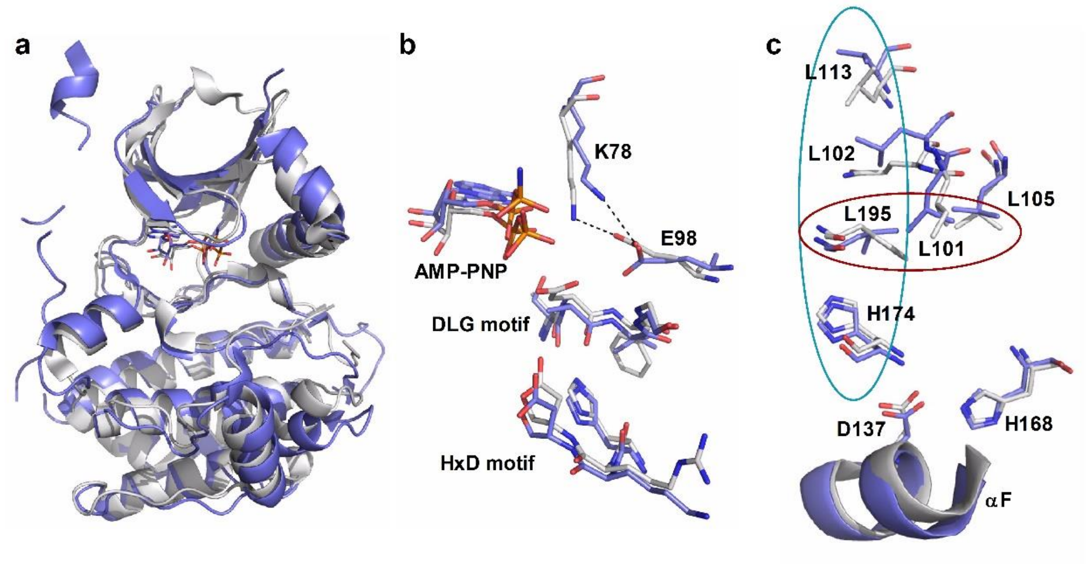

Fig. S6 Structure comparison of LKB1 and Aurora A. *(a)* Comparison of overall structures of LKB1 (PDB code: 2WTK) and Aurora A (PDB code: 1MQ4). *(b-c)* Although the active regulation mechanism of LKB1 is unique, the pattern of the catalytic core, the regulatory spine and the hydrophobic hook at the N-terminus of the activation segment are all conserved in the active conformation of LKB1. The carbons of LKB1 and Aurora A are colored purple and gray, respectively. The regulatory spine and the hydrophobic hook at the N-terminus of the activation segment were shown as blue and red ellipses, respectively. To avoid confusion, only residues of LKB1 are labeled.

Table S1. High resolution active structures used for alignment.

PDB code	Kinase	Resolution
3F66	c-Met	1.4
3A7F	STK24	1.5
2EU9	CLK3	1.5
2PVR	CK2	1.6
1JKL	DAPK	1.6
2QO7	Eph3	1.6
1QPC	Lck	1.6
3IDB	PKA	1.6
3S95	LIMK1	1.6
1IA8	Chk1	1.7
1O6K	PKB	1.7
3LXL	Jak3	1.7
1MQ4	Aurora A	1.9
4WR9	EGFR	1.9
1IR3	IRK	1.9
3KF4	Abl	1.9
2XIK	YSK1	1.9
3FE3	MAPK3	1.9
3BRB	MER	1.9
2J7T	STK10	2.0
2QLU	ACTR-IIB	2.0
2HK5	HCK	2.0
1K3A	IGF1RK	2.1
1HOW	SKY1	2.1
2J51	SLK	2.1
1QMZ	CDK2	2.2
1DAW	CK2	2.2
3COK	PLK	2.2
3COM	MST1K	2.2
3PLS	RON	2.2
2VZ6	CAMK2A	2.3
1CKJ	CK1	2.4
2ERK	ERK2	2.4
1O9U	GSK-3β	2.4
1CM8	P38	2.4
3L8P	Tie2	2.4
1K9A	Csk	2.5
2ZV7	Lyn	2.5
2PHK	Phk	2.6
EGFR	1M17	2.6

2ETR	ROCK1	2.6
1H4L	CDK5	2.7
2DQ7	Fyn	2.8
2VD5	DMPK	2.8
